# Predictors of a negative labour and birth experience based on a national survey of Canadian women

**DOI:** 10.1186/s12884-016-0903-2

**Published:** 2016-05-18

**Authors:** Andrei Smarandache, Theresa H. M. Kim, Yvonne Bohr, Hala Tamim

**Affiliations:** School of Kinesiology and Health Science, York University, 4700 Keele Street, Toronto, ON M3J 1P3 Canada; Department of Psychology, York University, 4700 Keele Street, Toronto, ON M3J 1P3 Canada

**Keywords:** Birth, Labour, Canada, Maternity experiences survey

## Abstract

**Background:**

A negative birth experience has been shown to have a significant impact on the well-being and future choices of mothers. The objective of this study was to assess the prevalence of, and identify the risk factors associated with a negative birth experience for women in Canada.

**Methods:**

The study was based on secondary data analysis of the Maternity Experiences Survey (MES), a Canadian population database administered to 6,421 Canadian women in 2006. The examined outcome - negative birth experience – was derived from mothers’ self-report of overall labour and birth experience. Independent variables were maternal demographics, health characteristics, pregnancy-related characteristics, and birth characteristics. Multivariable logistic regression analysis was performed to determine the significant predictors of negative birth experience. Adjusted Odds Ratios (AOR) and 95 % Confidence Intervals (CI) are reported.

**Results:**

Negative birth experience was reported among 9.3 % of women. The main significant predictors of a negative birth experience included older age (AOR 2.29, 95 % CI, 1.03–5.07), violence experienced in the past two years (AOR, 1.62, 95 % CI, 1.21–2.18), poor self-perceived health (adjusted OR, 1.95, 95 % CI, 1.36–2.80), prenatal classes attended (adjusted OR, 1.36, 95 % CI, 1.06–1.76), unintended pregnancy (adjusted OR, 1.30, 95 % CI, 1.03–1.63), caesarean birth (AOR, 1.65, 95 % CI, 1.32–2.06), and neonate admission to intensive care (AOR, 1.40, 95 % CI, 1.08–1.82).

**Conclusion:**

Significant predictors of a negative labour and birth experience were identified through this study, a first in the Canadian context. These findings suggest future research directions and provide a basis for the design and evaluation of maternal health policy and prevention programs.

## Background

A negative birth experience can impact women’s health and her offspring’s development well beyond the period of labour and birth. A negative birth experience has been associated with lower quality of life, lower self-rated health, persistent memory of pain, and the development of posttraumatic stress disorder (PTSD) or its symptoms [1–10]. One recent study found that subjective birth experience was the most important predictor of traumatic stress symptoms [11]. Of particular concern is the effect of a negative birth experience on the development of a fear of childbirth, which has been associated with a number of adverse outcomes including an increased incidence of caesarean birth [12–14], fewer future pregnancies [15] and postpartum depression [16]. Another study found that women with a previous negative birth experience showed significantly higher fear of childbirth than women who previously experienced two or more obstetric complications [17]. A Swedish study of 617 individuals found that 38 % of women who had a negative birth experience did not have additional children, versus 17 % of women reporting a positive experience (*p* < 0.05) [18]. Of the women who did have additional children, those with a negative birth experience had the subsequent child 4.2 years later versus 2.4 years later for those who had a positive experience [18].

A negative birth experience has also been found to be associated with increased likelihood of request for caesarean birth [19–23]. In Canada, the proportion of women with caesarean births has increased from 18.7 % (in 1997) to 27.3 % (in 2013) [24]. This may lead to decreased postnatal health and well-being [25], neonatal and maternal complications [26], and a negative birth experience [6, 19, 20]. It is also a predictor of development of PTSD [27], which in turn can also have significant repercussions for infant mental health. Furthermore, an increase in caesarean births were associated with maternal requests [23, 28]. Women who had previously given birth through caesarean section were more likely to prefer caesarean births again [23, 28], even in the absence of any clinical reasons [23]. A review of literature by McCourt and colleagues have also found that aside from clinical reasons, the increase in caesarean births were related to psychological factors, (such as poor care, perceived inequalities in care, fear about giving birth), cultural and social factors (such as auspicious birth dates, association with higher social and economic status), and the perception that caesarean births are a safer option [23].

Non-Canadian studies have estimated that between 10 and 20 % of women rate their birth experience as negative [29–31]. According to a comprehensive systematic review and non-Canadian studies, birth experience appears to be affected by a number of factors such as: fulfilled expectations [18, 30, 32], availability and quality of support, relationship between caregiver and patient, involvement in decision-making [29, 32–34], having the opportunity to be with the baby immediately after birth [33], unexpected complications during labour and birth [6], pain during labour and birth [29, 33, 34], and the ability to exercise personal control [6, 30, 33–35] which can also mediate the effects of pain [36]. Of these, the four factors – fulfilled expectations, support, relationship between caregiver and patient, and involvement in decision-making – were more predictive of birth experience than others such as pain, medical intervention, and birth setting [32].

Previous studies in the United States and Sweden have examined the association between birth experience and maternal demographics, health, pregnancy-related, and birth characteristics, sometimes with conflicting results [30, 35, 37]. For example, one study found a relationship between level of education and childbirth satisfaction [38], while another did not [30]. Income and mother’s age also show ambiguous effects [35]. Although factors such as fulfilled expectations, support, relationship between caregiver and patient, and decision-making were strong predictors of birth experience, other epidemiological factors such as immigration, ethnicity, violence experienced within the last 2 years were not examined in the Canadian population. Additionally, it is questionable whether education, income, and mother’s age are predictors of negative labour and birth experience in the Canadian population. The Maternity Experiences Survey (MES) is comprised of data collected among women across Canada with regard to their pre-pregnancy, pregnancy, and labour and birth experiences. The objective of this study was to assess the prevalence of negative birth experience for Canadian women and identify associated risk factors. To our knowledge, this initiative is novel as it is the first to query the predictors of negative labour and birth experience using a nation-wide data set of mothers in Canada. Findings will provide support to the design and implementation of policies and programs for sub-sets of vulnerable mothers, a strategy of particular importance in a population as diverse as Canada.

## Methods

### Database

The design of this study was cross-sectional. The analysis of the predictors of a negative labour and birth experience was based on data collected through the Maternity Experiences Survey (MES), designed by the Public Health Agency of Canada in partnership with Statistics Canada as part of the Canadian Perinatal Surveillance System. The survey included over 300 questions on pregnancy, birth, and the postpartum period. The target population consisted of mothers 15 years of age or older who had a singleton live birth between February 15, 2006 and May 15, 2006 in any Canadian province and November 1, 2005 and February 1, 2006 in any territory, and lived with their baby at the time of the survey. Mothers living on First Nations reserves or in institutions were excluded. A stratified random sample of 8,542 women was selected from the 2006 Canadian Census of Population and 6,421 eligible women (75.2 %) provided a response. Where possible, the questionnaire was provided in the mother’s preferred language. These respondents were weighted to represent 76,508 individuals and thought to be representative of the population for all characteristics examined. The survey was carried out through a telephone questionnaire by professional female interviewers with 96.9 % of the interviews held at five to nine months postpartum. The MES research protocol was reviewed by the Health Canada’s Science Advisory Board and Research Ethics Board and the Federal Privacy Commissioner, and approved by the Statistics Canada’s Policy Committee. Ethics approval was not needed as this was based on a secondary analysis of the MES collected by Statistics Canada. Access to the MES database was obtained through the Research Data Centre in Toronto, approved by Social Sciences and Humanities Council of Canada. Additional details on MES methods can be found in Dzakpasu et al. (2008).

### Outcome variable

The outcome variable of interest was self-rated *negative labour and birth experience*. Data were analyzed based on responses to one question in the MES that required the mother to rate her overall labour and birth experience. Responses were provided on a 5 point Likert scale which ranged from “very positive” to “very negative” labour and birth experience. For the purposes of this analysis, the labour and birth experience outcome variable was defined by combining responses of “somewhat negative” and “very negative” into one “negative” level, and responses of “neither negative nor positive”, “somewhat positive”, and “very positive” into one “non-negative” level.

### Predictors

Based on data availability and results of previous research, a number of variables were investigated as possible predictors of a negative labour and birth experience. These included: *maternal demographics* (mother’s age, urban-rural residence, immigration status, Aboriginal status, level of education, and partner status); *maternal health characteristics* (mother’s perceived health, smoking status during pregnancy, alcohol use during pregnancy, drug use during pregnancy, work status during pregnancy, and violence experienced in the past two years); *pregnancy related characteristics* (number of past pregnancies, prenatal classes attended, intended status of pregnancy, and health problems during pregnancy); and *labour and birth characteristics* (type of birth, setting of the baby’s birth, care provided in a language the mother understood, needed to travel for the birth, birth of baby attended by the family doctor, and whether the baby had to be interned in an intensive/special care unit). All of these variables were self-reported by the mother [39].

### Statistical analysis

The prevalence of negative labour and birth experience was investigated at the national level and by province and territory. To assess the relationship between different predictors and negative labour and birth experience, chi-square tests and odds ratio (OR) were calculated using cross tabulations and logistic regression. A multivariable logistic regression model was performed with all potential predictor variables being considered as independent variables and negative labour and birth experience variable as the dependent variable. To account for complex sampling design, bootstrapping was performed where appropriate to calculate all the OR and 95 % confidence interval (CI) estimates. Population weights, normalized weights, and bootstrap weights were all created by Statistics Canada and provided with the MES data set. The sample sizes reported in this manuscript were derived using normalized weights, weighted to represent a larger population. All analyses were computed with Stata Data Analysis and Statistical Software (version 13.0), and set at alpha <0.05 for two-tailed test for statistical significance.

## Results

The MES sample size of 6,421 respondents, weighted to represent 76,508 women was analyzed in this study. Of the 6,421 respondents, 6,384 provided a complete response to the MES question asking the mother to rate her overall labour and birth experience. Of the mothers who responded to this question, 53.8 % rated their birth experience as very positive (*n* = 3,437), 26.2 % as somewhat positive (*n* = 1,647), 10.7 % as neither negative nor positive (*n* = 685), 6.2 % as somewhat negative (*n* = 395), and 3.1 % as very negative (*n* = 196). In total, 9.3 % of the women surveyed who responded to this question rated their labour and birth experience as somewhat negative or very negative (*n* = 591).

**Fig. 1:**
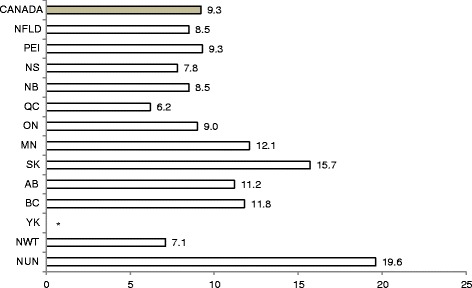
Distribution of women’s ratings of negative labour and birth experience by province/territory, Canada, 2006–2007 (%). * Estimate not shown due to cell count less than 5

Figure 1 illustrates the distribution of women who reported a negative labour and birth experience by province or territory. This figure varies widely by region, with a low of 6.2 % in Quebec and a high of 19.6 % in Nunavut. Women in the Atlantic Provinces generally reported lower rates of negative birth experience, ranging from 7.8 % in Nova Scotia to 9.3 % in Prince Edward Island, than women in Western Canada, ranging from 11.2 % in Alberta to 15.7 % in Saskatchewan. At 9.0 %, the proportion in Ontario is close to the national average.

Table 1 provides an overview of the results of the association between the independent variables (*maternal demographic, health, pregnancy, and birth characteristics*), and negative labour and birth experience. Results of the multivariable logistic regression model showed age to be the only variable among the list of maternal demographic characteristics studied to be significantly associated with negative labour and birth experience. Mothers who were 30-39 years of age and 40 years and older were respectively 1.90 times (95 % CI, 1.08-3.34) and 2.29 times (95 % CI, 1.03-5.07) more likely to experience negative labour and birth experience compared to mothers who were younger than 20 years old after adjusting for other variables.

**Table 1:** Prevalence and predictors of a negative labour and birth experience based on a national survey of Canadian women

Predictors	Sample size *N* ^a^	Negative experience	Unadjusted odds ratio		Adjusted odds ratio	
		*N* ^b^ (%)	OR^c^	(95 % CI)^c^	OR^c^	(95 % CI)^c^
*Maternal Demographics*						
Age (years)						
<20	188	22 (11.5)	1		1	
20–29	2941	238 (8.1)	0.68	0.46–1.00	1.42	0.84–2.40
30–39	3033	306 (10.1)	0.86	0.59–1.27	1.90	1.08–3.34
> = 40	189	20 (10.5)	0.91	0.48–1.73	2.29	1.03–5.07
Urban-rural residence						
Rural area	1100	84 (7.6)	1		1	
Urban, population ≤499,999	2282	222 (9.7)	1.31	1.01–1.70	1.25	0.94–1.65
Urban, population ≥500,000	2781	259 (9.3)	1.25	0.95–1.63	1.22	0.91–1.65
Immigration to Canada						
No	4960	448 (9.0)	1		1	
Yes	1396	141 (10.1)	1.13	0.91–1.42	1.05	0.80–1.39
Ethnicity						
Non-Aboriginal	6087	563 (9.3)	1		1	
Aboriginal	267	25 (9.4)	1.01	0.68–1.52	0.82	0.51–1.31
Level of education						
High school or less	1321	129 (9.8)	1		1	
Some postsecondary education	2752	241 (8.7)	0.88	0.70–1.11	0.89	0.68–1.16
Undergraduate education	1626	154 (9.5)	0.96	0.75–1.24	0.91	0.67–1.25
Graduate education	623	63 (10.1)	1.03	0.74–1.43	1.01	0.68–1.49
Partner/Significant other						
No	528	57 (10.7)	1.20	0.90–1.60	1.08	0.76–1.53
Yes	5832	532 (9.1)	1		1	
*Maternal Health Characteristics*						
Moms perceived health						
Excellent/very good	4628	381 (8.2)	1		1	
Good	1413	156 (11.1)	1.39	1.13–1.69	1.24	0.98–1.56
Poor/Fair	340	53 (15.6)	2.07	1.50–2.84	1.95	1.36–2.80
Cigarette smoking during pregnancy						
No	5705	520 (9.1)	1		1	
Yes	667	69 (10.4)	1.15	0.88–1.51	1.00	0.72–1.40
Alcohol use during pregnancy						
No	5681	546 (9.6)	1		1	
Yes	664	44 (6.6)	0.66	0.47–0.92	0.69	0.48–1.00
Drug use during pregnancy						
No	6307	582 (9.2)	1		1	
Yes	59	7 (11.7)	1.31	0.54–3.16	1.38	0.53–3.59
Work during pregnancy						
No	1966	183 (9.3)	1		1	
Yes	4392	406 (9.2)	0.99	0.82–1.21	1.02	0.81–1.28
Experienced violence within last 2 years						
No	5657	494 (8.7)	1		1	
Yes	693	92 (13.2)	1.59	1.24–2.05	1.62	1.21–2.18
*Pregnancy-Related Characteristics*						
Number of past pregnancies						
None	2890	305 (10.6)	1.34	1.12–1.60	1.11	0.86–1.45
1 or more	3474	282 (8.1)	1		1	
Attended prenatal classes						
No	4295	359 (8.4)	1		1	
Yes	2085	231 (11.1)	1.37	1.14–1.64	1.36	1.06–1.76
Intended status of pregnancy						
Then or Sooner	4622	403 (8.7)	1		1	
Later or Not at all	1713	182 (10.6)	1.24	1.02–1.51	1.30	1.03–1.63
Health problems during pregnancy						
No	4821	401 (8.3)	1		1	
Yes	1556	188 (12.1)	1.51	1.25–1.82	1.43	1.16–1.76
*Birth Characteristics*						
Type of birth						
Vaginal	4708	366 (7.8)	1		1	
Caesarean	1676	224 (13.4)	1.83	1.52–2.21	1.65	1.32–2.06
Birth setting						
Hospital/clinic	6250	585 (9.4)	2.61	0.76–9.01	1.75	0.50–6.10
Birthing centre/private/other	134	5 (3.8)	1		1	
Care provided was in language understood						
No	183	27 (14.8)	1		1	
Yes	6170	561 (9.1)	1.13	0.93–1.38	1.21	0.96–1.51
Had to travel elsewhere for birth						
No	4744	426 (9.0)	1.74	1.09–2.77	1.41	0.78–2.58
Yes	1637	165 (10.1)	1		1	
Healthcare provider attended birth						
Family doctor	5447	505 (9.3)	1.22	0.92–1.61	0.95	0.71–1.28
Other	792	61 (7.8)	1		1	
Baby in intensive/special care unit						
No	5563	484 (8.7)	1		1	
Yes	810	105 (12.9)	1.56	1.23–1.96	1.40	1.08–1.82

^a^Sample size is estimated using normalized weights

^b^Frequencies are row percentages estimated using normalized weights

^c^OR and 95 % CI were calculated using bootstrapping technique

The mother’s perceived health, her alcohol use during pregnancy, and whether she experienced violence in the past two years were the three (unadjusted) maternal health characteristics that were significantly associated with negative labour and birth experience. At the multivariable regression level, only the mother’s perceived health and experienced violence remain significant. Women whose perceived health were poor or fair were significantly more likely to report a negative birth experience (adjusted OR, 1.95, 95 % CI, 1.36-2.80) than those who reported excellent or very good health. Furthermore, having experienced intimate partner violence in the past two years was associated with a higher incidence of a negative labour and birth experience (adjusted OR, 1.62, 95 % CI, 1.21-2.18).

The pregnancy-related characteristics that were significantly associated with negative labour and birth experience at the unadjusted level were the number of past pregnancies, attendance at prenatal classes, intended status of pregnancy, and any health problems experienced during pregnancy. At the multivariable level, significance remained for those who attended prenatal classes (adjusted OR, 1.36, 95 % CI, 1.06–1.76), those with unplanned or unwanted pregnancy (adjusted OR, 1.30, 95 % CI, 1.03–1.63) and those who reported having experienced health problems during pregnancy (adjusted OR, 1.43, 95 % CI, 1.16–1.76).

Of the birth characteristics considered at the bivariate level, type of birth, not having to travel somewhere else for the birth, and admission of the neonate to an Intensive Care Unit (ICU) predicted a negative labour and birth experience. However, at the multivariable level, only those mothers who had a caesarean birth (adjusted OR, 1.65, 95 % CI, 1.32–2.06) and mothers whose babies were interned in an ICU (adjusted OR, 1.40, 95 % CI, 1.08–1.82) were significant.

## Discussion

The present study aimed to identify the demographic, health, pregnancy, and birth related characteristics associated with negative labour and birth experience among women in Canada. On average, 9.3 % of Canadian mothers rated their birth experience as somewhat negative or very negative, with the highest averages reported in Western Canada. Most notably, negative labour and birth experience among these mothers was significantly associated with older age, poorer perceived health, domestic violence, prenatal classes, and birth by caesarean section. The findings are novel in that this was the first study to identify the risk factors associated with negative birth and labour experience among Canadian mothers using a national database.

Based on the analysis of this national sample, the proportion of Canadian women who report a negative birth experience (9.3 %) was similar to findings in non-Canadian studies, which have estimated that between 10 % (Sweden) and 20 % (the Netherlands) of women rate their labour and birth experience as negative [29–31]. Mothers over the age of 30 years were two times more likely to report a negative experience compared to younger mothers. It is found that older women perceive labour and birth differently than their younger mom counterparts however, their own birth experience does not necessarily translate into being negative [40]. The study results are found to be in contrast to previous studies that did not find a significant relationship between older age and birth experience [6, 30, 41]. The relationship identified in the present study may be partly explained by the increased incidence of birth complications in older mothers [42, 43], as well as the likelihood of having time to develop specific expectations for their birth care, experience, relationship, and involvement in decision making that are not being met – that may ultimately affect their negative birth experience. The differences among the studies may be attributed to variations in study design, divergent variable definitions, and sample selection. Therefore, further research is required to elucidate the specific reasons for the present findings.

In this study, mother’s poor perceived health and intimate partner violence experienced at home were the only significant maternal health characteristics of negative labour and birth experience. The outcomes associated with poor perceived health are in accordance with the findings among primiparous mothers in Sweden, where negative birth experience after operative birth was associated with poor self-rated health soon after birth and up to 1 year after childbirth [2]. Although perceived health was self-reported by the mother, the association between self-rated health and mortality, morbidity, hospital use, and physician contact is well-established [44–46]. However, further investigation into how poor perceived health generally contributes to negative birth experiences is warranted as factors such as obesity [47], pre-eclampsia [48], and depression [49], which have been commonly reported during pregnancy and birth can be both or either a predictor and a result. With regard to experienced violence, systematic reviews have found that women who have experienced abuse are significantly more likely to encounter adverse birth outcomes such as higher infant mortality, low birth weight, and preterm births [38, 50]. This association, along with the lack of social support that may accompany an abusive relationship, may explain the reason why women who have experienced violence are more likely to rate their birth experience as negative.

Among pregnancy-related characteristics, attending prenatal classes was found to be one of the more surprising factors found to be significantly associated with negative labour and birth experience. Indeed, in this sample, attending prenatal classes was found to increase the risk of negative labour and birth experience. However, inconsistencies have been reported in previous research that produced similar results [6, 51]. It can be speculated that the content and delivery of the prenatal classes (i.e., support available, size of the classes, duration, and frequency) may vary widely across prenatal centres that may explain the variability among different studies. Some prenatal classes may allow the mother to feel fulfilment of expectations and a sense of control both of which have been documented to contribute to a positive birth experience [18, 30, 41], whereas other classes may not meet such expectations. Future research could explore the content of prenatal classes with a view toward identifying whether the curriculum and mode of delivery meet realistic expectations and increase a sense of control for mothers-to-be.

The most notable birth characteristic related to negative labour and birth experience was the type of birth (i.e., vaginal or caesarean). Previous studies have also found a significant relationship between caesarean birth and lower childbirth satisfaction [6, 13, 30]. This is unsurprising given that caesarean sections are often associated with unexpected complications during childbirth. Experiencing such complications make the overall labour and birth experience as negative for the mother where they may report feelings of loss (i.e., of rite of passage of body capability or of womanhood), fear, as well as physical pain following operative birth [2, 6, 52, 53]. Furthermore, these complications during childbirth may result in greater likelihood for the newborn to be admitted to the Intensive Care Unit (ICU). Our findings show that the admittance of the newborn baby to the ICU may be deemed as deleterious to the birth experience, as having an opportunity to be with the baby immediately after birth enhances the birth experience [33].

In the current study, several potentially predictive characteristics did not contribute to negative labour and birth experience. Smoking, alcohol use, and drug use were not found to be predictors of a negative birth experience after adjusting for other variables. In terms of smoking, a similar conclusion was reached in previous research [6]. To our knowledge, the question of whether alcohol and drug use are related to mother’s birth experience has not been recently explored. Interestingly, although the harm to the infant of the use of alcohol and some recreational drugs is well-documented [54–56], using these substances does not seem to affect how mothers perceive their labour and birth experience. It is postulated that alcohol and other substance use may contribute to relaxation or a more laid back approach of birth experience in some mothers. Alternatively, this may in part be explained by a limitation of the present study, in which the variables analyzed were coded to indicate whether the mother has ever used any of these substances in the 12 months before giving birth, without identifying the frequency or intensity of use. A possible future direction for research is to determine whether heavy users of these substances are more likely to report a negative birth experience.

Interpretation of this study is subject to a number of limitations. All of the variables included in this model, including the outcome variable of *labour and birth experience*, were based on self-report of participants. As such, some variability likely exists between how different individuals defined their experience. However, self-report measures have proven to be just as effective in predicting health outcomes as documented in several studies [44–46]. Because this was based on a secondary data analysis of a population-based survey collected by Statistics Canada, the availability of variables were limited and thus our study was not able to evaluate data that described the context of care (e.g., relationship with providers, active involvement in decision-making, and fulfillment of expectation). Further, the cross-sectional nature of the study made it difficult to infer causation between the outcome variable and its predictors. However, despite these limitations, this study represents the first comprehensive analysis of the predictors of a negative labour and birth experience among Canadian women.

## Conclusion

The findings of this study may be useful in guiding maternal health policy by identifying areas of focus for innovative prevention and intervention programs that focus on perinatal maternal mental health. For example, caesarean sections are increasing among women in Canada [24], which becomes an important factor to consider when examining its relationship to labour and birth experience. The may also be helpful in informing a re-evaluation of current policies and programs to improve mother’s birth experiences in view of achieving healthy maternal and child outcomes. The risk factors identified in this study should also be further researched in terms of *how* and *why* they influence the birth experience. This could be accomplished through qualitative studies such as personal interviews and narratives with individual mothers.

### Ethics approval and consent to participate

The MES research protocol was reviewed by the Health Canada’s Science Advisory Board and Research Ethics Board and the Federal Privacy Commissioner, and approved by the Statistics Canada’s Policy Committee. Ethics approval was not needed as this was based on a secondary analysis of the MES collected by Statistics Canada. Access to the MES database was obtained through the Research Data Centre in Toronto, approved by Social Sciences and Humanities Council of Canada.

### Consent for publication

The MES survey is voluntary. Implicitly, participation in a voluntary survey requires consent. Respondents are informed of the voluntary nature of the survey through a notice prior to the start of the data collection. Interviewers are also instructed to permit respondents to refuse to answer any question or to terminate an interview at any time. Statistics Canada is prohibited by law from releasing any data that relates to any identifiable person without prior knowledge or the consent in writing of that person. The MES database does not include personal identifiers such as name, address, and telephone number. Various confidentiality rules are applied to the MES data that are released or published to prevent the publication of any confidential information. Data are suppressed to prevent direct or residual in order to protect the identity of the participants. For further information, please see: http://www.statcan.gc.ca/eng/rdc/mitigation

### Availability of data and materials

The data will not be made available in order to protect the participants’ identity. This study was based on a secondary analysis of the MES database collected by Statistics Canada, which requires the researcher to submit an application to access the data at a Research Data Centre. Access to the MES database was obtained through the Research Data Centre in Toronto, approved by Social Sciences and Humanities Council of Canada. The data is available through submitting a formal application to the Research Data Centre in Canada.
